# Measurement of Nasal Contour Landmarks in Septorhinoplasties with Special Regard to the Course of Postoperative Swelling Using a Three-Dimensional Camera

**DOI:** 10.3390/life14070813

**Published:** 2024-06-27

**Authors:** Katharina Storck, Julia Eufinger, Sebastian Kotz, Carolina Classen, Lucas M. Ritschl

**Affiliations:** 1 Department of Otolaryngology, Head and Neck Surgery, Klinikum Rechts der Isar, School of Medicine and Health, Technical University Munich, 81675 Munich, Germany; katharina.storck@mri.tum.de (K.S.); sebastian.kotz@mri.tum.de (S.K.); 2 Department of Oral and Maxillofacial Surgery, School of Medicine, University of Saarland, Homburg/Saar, 66421 Homburg, Germany; carolina.classen@tum.de; 3 Department of Oral and Maxillofacial Surgery, Klinikum Rechts der Isar, School of Medicine and Health, Technical University Munich, 80333 Munich, Germany; lucas.ritschl@tum.de

**Keywords:** 3D photography, rhinoplasty, 3D technologies, post-operative swelling behavior

## Abstract

The integration of three-dimensional (3D) cameras into clinical practice for pre-operative planning and post-operative monitoring of rhinoplasties remains controversial. However, this technology offers the advantage of capturing the 3D surface without exposing patients to potentially harmful radiation. Continuous assessment allows the follow-up of swelling patterns, cartilage alignment, and bone remodeling. The primary objective of our study was to quantify changes in nasal structure before and after rhinoplasty by using 3D photography. Our study cohort consisted of 29 patients who underwent open structural rhinoplasty. We used the Artec Space Spider camera to acquire a total of 103 3D images. We collected pre-operative and at least two or three post-operative follow-up scans, which were taken one, three, and six months after surgery. We evaluated paired scans that included various time intervals to improve our understanding of swelling behavior and to ensure an objective analysis of changes. Eleven specific anatomical landmarks were identified for measurement. Two independent raters determined the distances between these landmarks over time. The calculation of intraclass correlation coefficients showed low inter-rater variability. Statistically significant changes over time (*p* < 0.05) were observed for various anatomical landmarks, including soft tissue nasion, soft tissue orbitale right, soft tissue maxillofrontale left, soft tissue maxillofrontale right, nasal bridge, and nasal break point. Conversely, no significant changes (*p* > 0.05) were observed in the measurements of soft tissue orbitale left, pronasale, subnasale, alare right, or alare left. A visual assessment was conducted using surface distance maps. The results indicate that the complete decrease in swelling takes at least 6 months or even longer. Additionally, 3D photography can provide an objectively comparable analysis of the face and external contours. Furthermore, it allows for a comparison of external contours and therefore pre- and post-operative differences.

## 1. Introduction

The current state-of-the-art imaging techniques in rhinosurgery are two-dimensional (2D) photography and 2D cephalometry to document, distinguish, and analyze the pre- and post-operative nasal state [1]. Shadows on the face, different imaging angles, and diurnal light variations can complicate a standardized analysis of the nose and face. Three-dimensional (3D) photography, in contrast, can provide an objectively comparable analysis of the face and external contours and can measure the comparability of external contours and pre- and post-operative volume differences [2]. Nevertheless, the value of and need for 3D cameras in clinical practice and in the post-operative follow-up of rhinoplasties remain under discussion, despite it offering the possibility of additional irradiation-free imaging with improved visualization and comparability [3].

In addition, multiple consecutive measurements after various time intervals allow the assessment of swelling behavior, cartilaginous apposition, and bony remodeling. Determination of contour deviations is enabled by the superimposition of two corresponding geometries and measurements. Concerning the operation performed (in our case the rhinoplasty), the areas with relevant changes in external shape can be identified and evaluated regarding the desired change in the shape.

Communication with the patient, surgical planning, and the visualization/calculation of pre- and post-operative shape and volume changes can be performed more easily from every angle of the face [3]. Three-dimensional camera systems are considered a valid method for objectively comparing surgical procedures in clinical practice [4] and have therefore become an important tool in this field.

The literature only mentions the analysis of the variations of volume without the assessment of specific anatomical landmarks [5]. There is no published study on septorhinoplasty that is adequately comparable to the current investigation. The aim of this study was therefore to objectify pre- and post-operative changes of the nose after rhinoplasty using 3D scans, and to determine whether changes in the shape and swelling of the nose persist up to 6 months after surgery using specific landmark measurements. Understanding these adaptations during the healing phase can enhance the management of patient expectations and improve surgical techniques, leading to optimal aesthetic and functional outcomes.

## 2. Materials and Methods

The study complies with the current Declaration of Helsinki and was reviewed and approved by the Ethics Committee of the Klinikum rechts der Isar of the Technical University of Munich (2022-347-S-KK). All patients were consecutively recruited in the Department of Otorhinolaryngology, Head and Neck Surgery within a period of 12 months and received a septorhinoplasty independently of the study. All patients were informed about the procedure of the study and the anonymized use of their data, and written informed consent was obtained. A separate consent form was signed for each image shown in this paper. Neither the authors nor any of the participants of the study benefitted financially or economically, and no conflicts of interest arose.

### 2.1. Patient Population

Voluntary participation within this prospective study was offered to all patients irrespective of gender receiving a functional and aesthetic septorhinoplasty at the Department of Otorhinolaryngology, Head and Neck Surgery, Klinikum rechts der Isar of the Technical University of Munich (Table 1). Patient commitment was ensured through regular post-operative follow-ups. Further inclusion criteria were the lack of epileptic disease and the adult age of the patients. Exclusion criteria were revision rhinoplasty with/without rib cartilage, the use of fillers, botox or hyaluronic acid and the use of a piezotome.

### 2.2. Surgical Treatment Procedure

All patients received an open structural rhinoplasty including osteotomies, all being performed by the same surgeon (KS). Depending on the pathology of the cartilaginous and osseous framework of the nose, well-known techniques were performed to achieve the desired outcome of the surgery. Post-operatively, the rhinoplasty was stabilized with internal and external splinting. The inner septal splints include an integrated breathing tube and were usually left in place for five days. External splinting included steristrips applied in the manner of roof tiles, and a nasal cast was adapted to them. All patients received the same cooling protocol with the Hilotherm system (Hilotherm^®^ by Hilotherm group GmbH; Argenbühl-Eisenharz, Allgäu, Germany) for 48 h. After one week, the external splinting was always replaced by a new one. Compression of the nose by steristrips was continued by the patient after removal of the nasal cast for at least two months post-operatively.

### 2.3. Camera System

In a previous observational study, we compared three-dimensional imaging of the nose using three different 3D-photography systems. Due to the results, we decided to use the Artec Space Spider camera in this study [6].

The Artec Space Spider camera is a 3D scanner (Artec 3D, L-1748 Senningerberg, Luxembourg) based on blue light technology specifically designed for CAD (computer-aided design) and produces a 3D image. It projects a mesh of structured light onto the scanned geometry and creates a 3D framework based on the distortion of the mesh [7]. The portable camera is operated manually and can be moved and used as desired because of its light weight (0.85 kg) [8]. Its accuracy is stated as being up to 0.05 mm, with a resolution of up to 0.1 mm [7,8]. The data acquisition rate is 1 million points per second. The working distance is 0.2–0.3 m [8]. Once the image has been acquired within approximately 2–5 min, it is processed in the associated software [7]. The connection of a rechargeable battery allows independence in use [9]. The scanning of dry and wet surfaces is also possible, with only a minimal decrease in accuracy on wet surfaces [10]. In our study, scanning was always performed under the same dry conditions.

### 2.4. Data Acquisition and Measurements

Following a pre-operative scan, two or three post-operative 3D photographs were taken from each patient with the following post-operative intervals: one month, three months (±two weeks), and six months (±two weeks). To obtain an optimal scanning result, lighting in the consulting room was slightly dimmed, and the patient was always positioned under the same light conditions within the same room. Before the actual scan was performed, the camera system was warmed up as recommended by the manufacturer. The protocol of the University of Sheffield was followed, as this was considered proper usage of the Artec Space Spider camera [11].

The patients were instructed to close their eyes. The face was then scanned from all angles until a homogeneous texture was obtained with complete coverage of the nose. First and foremost, the nose as the object of interest was scanned according to comparable studies [10]. Immediate feedback from the Artec Space Spider (Artec Studio 13.2.4.6 (18.10.2019)) Software—13 allowed a constant distance of the camera to the patient to be maintained. The patient’s facial expression had to be neutral to avoid the comparability of the 3D scans being compromised and the reproduction of a valid result being jeopardized [4]. During the scan, the 3D camera was connected to a high-performance laptop (HP ZBook 17 G4, Hewlett-Packard^®^, Palo Alto, CA, USA), which displayed the images in real-time.

Data processing and storage was performed using the associated Artec Studio Professionals software (version 13). Images were checked for artifacts, which were removed using the “*error*” function of the software. Then, the pre-processed 3D images were exported and saved as .stl (Standard Tessellation Language) files, which represent the universal interface in 3D photography. The generated .stl data sets of the face were subsequently pseudonymized, virtually analyzed, and compared. No post-processing within the respective software system or subsequent conversion by an external software program was carried out. This allowed comparable .stl data sets and prevented unforeseen data loss.

To objectify the changes of corresponding geometries, the following pairs were compared with each other: the pre-operative scan versus the scan at the 1st month, the scan taken at the 1st month versus that at the 3rd month, the pre-operative scan versus the scan at the 3rd month, the pre-operative scan versus the scan at the 6th month, and the scan at the 3rd month versus that at the 6th month. The distances between the defined anatomical landmarks in the 3D scans were calculated.

### 2.5. Anatomic Landmarks

Anatomically defined soft tissue points were selected to achieve the best possible and the most precise comparability, since muscle movements and breathing might influence the reproducibility [12]. Eleven soft tissue points were chosen to compare the scans.

**Figure 1:**
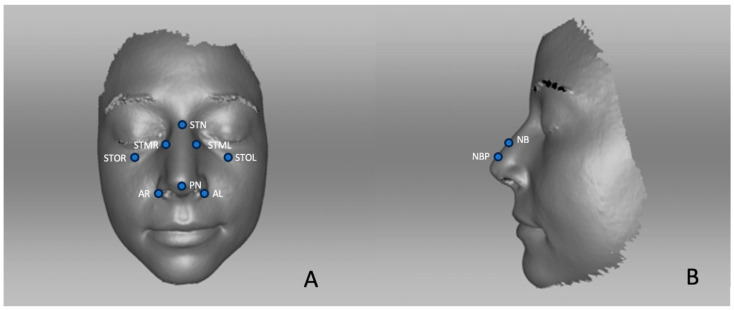
Visualized landmarks: (**A**) frontal view and (**B**) lateral view. STN (soft tissue nasion), STOL (soft tissue orbitale left), STOR (soft tissue orbitale right), STML (soft tissue maxillofrontale left), STMR (soft tissue maxillofrontale left), PN (pronasale), NB (nose bridge), NBP (nasal break point), AR (alare right), AL (alare left).

These landmarks include the soft tissue nasion (STN), an important point marking the base of the nasal root [13]. Soft tissue orbitale left (STOL) and right (STOR) mark the inferior level of the respective side of the infraorbital rim [13]. Soft tissue maxillofrontale left and right (STML and STMR) are situated at each lateral margin of the base of the nasal root [12]. Pronasale (PN) corresponds to the most anterior point of the nasal tip, whereas subnasale (SN) is the central point along the soft tissue contour passing from the columella ridge to the upper lip [13]. Nose bridge (NB) represents the bridge of the nose [13]. The nasal break point (NBP) designates the supra tip area of the nose in the lateral view [13]. Alare right (AR) and alare left (AL) are the furthest lateral points on each alar contour [13]. The landmarks are shown in Figure 1. The descriptive statistics are given in mm, including mean, std. deviation, minimum, maximum, and the 25th, 50th, and 75th percentiles. A positive sign indicates an increase in distance. A negative sign represents a decrease in distance.

Significant variations over time were noted for numerous anatomical landmarks, such as STN, STOR, STML, STMR, NB, and NBP. Conversely, no noteworthy variations were seen in the measurements for STOL, PN, SN, AR, or AL.

All measuring points were determined twice by the same two raters (JE and CC), who were familiar with the use of the camera system and the program. Additionally, the intraclass correlation coefficient (ICC) was calculated to assess the reliability of the measurements.

### 2.6. Surface Distance Maps

Color-coded maps showed any differences between two superimposed scans of corresponding geometries. The change was displayed in mm. To identify the regions with the greatest change, the distance to search was set to 3 mm, ensuring that relevant changes could also be visualized in color on the map [14]. 

### 2.7. Statistical Analysis

Prior to patient recruitment, a case number calculation for the implementation of the study was performed using G-Power version 3.1 software (Axel Buchner, Universitätsstraße 1, 40225 Düsseldorf, Germany). The estimated effect size was 0.8, the α-error probability: 0.05, the power: 0.95, and the minimum case number: 19 patients. However, it was specified that at least 20 participants should be included. The initial data distribution was examined, and then a parametric *t*-test for paired data was performed for more detailed anatomical landmark analysis. Intraclass correlation coefficients were computed separately for each dependent variable and each time point by using a two-way mixed-effects model.

## 3. Results

In total, 29 patients were enrolled, including 20 females and 9 males (Table 1), and 103 3D scans were performed, analyzed, and compared.

**Figure 2:**
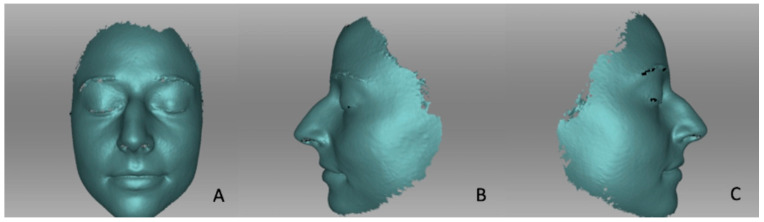
Pre-operative 3D photography: (**A**) frontal view, (**B**) lateral view left, (**C**) lateral view right.

**Figure 3:**
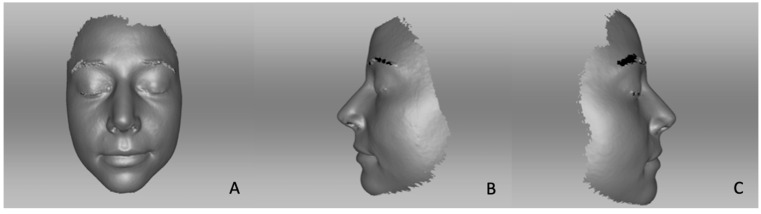
Three-month post-operative 3D photography (corresponding case to Figure 2): (**A**) frontal view, (**B**) lateral view left, (**C**) lateral view right.

Figure 2 shows exemplarily a pre-operative 3D scan (Figure 2). Similarly, images were obtained at one, three, and six months post-operatively (Figure 3). The images were then automatically aligned and subsequently measured. Nevertheless, the auto-alignment was controlled and checked for accuracy in every case. Afterwards, every point on the created mask of the patient was found in the 3D-coordinate network.

**Figure 4:**
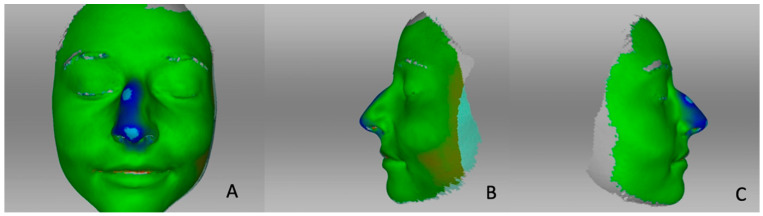
Surface distance maps comparing pre-operative and three-month post-operative 3D photography: (**A**) frontal view, (**B**) lateral view left, (**C**) lateral view right. Areas marked in blue are those that have lost substance. Areas in red have gained substance.

**Figure 5:**
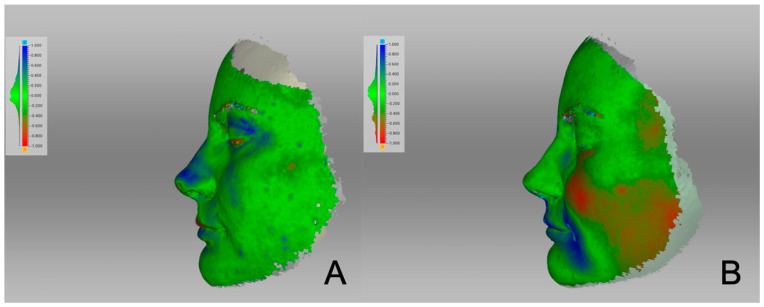
Surface distance maps analyzing changes over time: (**A**) comparison of one- and three-month post-operative 3D photography; (**B**) comparison of three- and six-month post-operative 3D photography. Areas marked in blue are those that have lost substance. Areas in red have gained substance.

The surface distance map clearly showed the areas that had changed (Figure 4). The nasal hump (marked in blue) was reduced with a desired rotation of the nasal tip. The areas that had lost substance were marked in blue. The face remained relatively unaffected by changes, with a slight increase in the left cheek area (marked in red). To compare the changes in a single patient between the initial three post-operative months and those three to six months later, surface distance maps are presented in Figure 5 (Figure 5). The figure illustrates that more changes occurred during the first three months after the operation than between months three and six.

### Landmark Analysis

**Figure 6:**
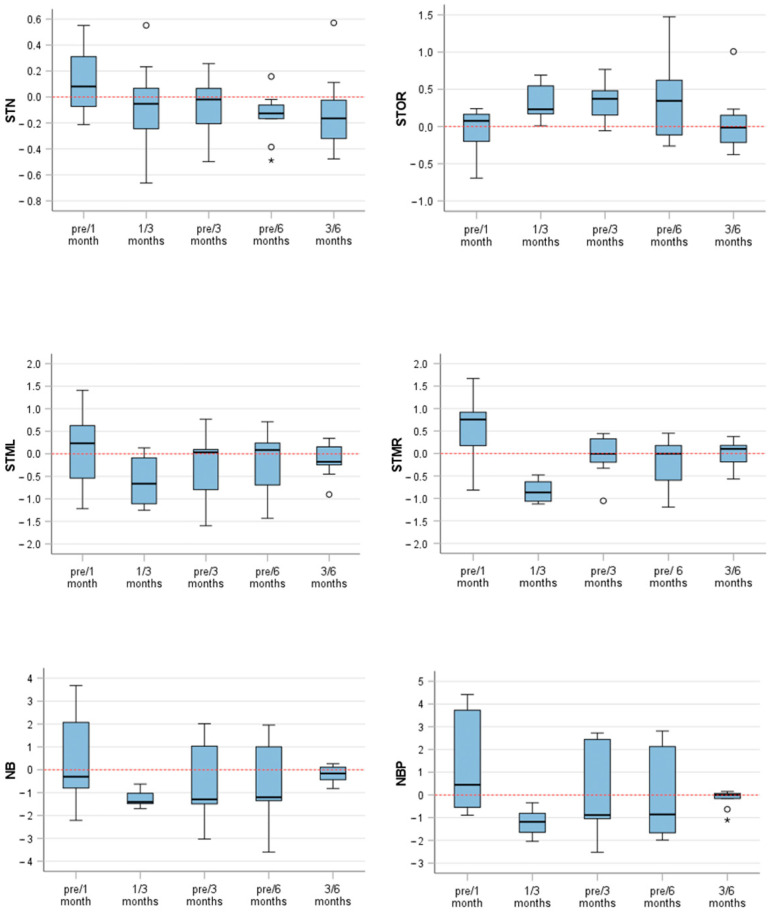
Box plots visualizing the descriptive statistics (mm) between the mentioned pairs and landmarks. STN (soft tissue nasion), STOR (soft tissue orbitale right), STML (soft tissue maxillofrontale left), STMR (soft tissue maxillofrontale left), NB (nose bridge), NBP (nasal break point). Circles represent mild outliers. Stars represent extreme outliers.

The results of landmark analysis are shown in (Table 2) and visualized in (Figure 6). The first pair of the NBP (NBP—pre/1 month vs. pre/3 months) showed one of the most prominent differences. The measurements displayed a significant average distinction of 1.296 units ± 0.825, indicating a highly significant contrast (*p* < 0.001) between the measurements. Additionally, another remarkable change was observed in the second pair (NBP—pre versus 3 months and pre versus 6 months), with a mean difference of 0.232 units ± 0.507. The statistical significance of the study was demonstrated by a t-value of 2.286 and a corresponding *p*-value of 0.031. Pair 3 (NBP—1 versus 3 months and 3 versus 6 months) showed a highly significant difference of −1.035 units between NBP measurements taken after 1–3 post-operative months and 3–6 post-operative months. The standard deviation was 0.575, and the t-value was −5.406, with a *p*-value of less than 0.001. The paired samples test results also provided *p*-values determining the level of statistical significance for differences in the measurement of the NB over variable time intervals. The results showed highly significant differences in pair 1 and pair 3, as indicated by their low *p*-value (*p* < 0.001).

Furthermore, paired sample tests were provided for other landmarks, such as STMR, STML, STOR, and STN. These tests identified variable levels of significance. Intraclass correlation coefficients were >0.9 for all anatomical landmarks, except for STN 3 versus 6 months (0.745).

## 4. Discussion

Septorhinoplasties are among the five most commonly practiced procedures in plastic surgery [15]. At the same time, they represent one of the most challenging procedures, as various psychosocial, functional, and aesthetic aspects must be taken into account [16]. The preparation for a septorhinoplasty procedure is highly challenging for the surgeon, as aesthetic and functional aspects have to be considered in the same way, congruent with the patient’s wishes [17]. Optimal outcomes require both the surgeon’s and the patient’s satisfaction [18], but ultimately, the success of septorhinoplasties is defined significantly by patient’s satisfaction [3]. 

Standard pre- and post-operative photographic documentation is normally performed using 2D cameras [1,3]. This includes frontal, lateral, hemifacial, and basal images [3], and laughing [19]. The visualization of a 3D object, such as the nose and the face within a 2D picture, cannot be achieved flawlessly. In brief, the representation of a 3D object within a 2D photograph is not possible without having to tolerate certain information losses [1,3].

Common complications of any facial surgery include pain, swelling, and loss of function. Maximum swelling is reached post-operatively after an average of 48–72 h [20]. Post-operatively, however, the nose undergoes remodeling over several months and affects bone, cartilage, and soft tissue. The data on the duration of the remodeling in septorhinoplasty are sparce. However, any statement about swelling behavior and remodeling holds out possibilities of determining the final time-point of therapy success and of being able to present study-based information to the patient on demand. In addition to pure swelling behavior and to bony and cartilaginous remodeling, the face might also be subject to diurnal volume changes. For this reason, our scans were performed under the same conditions within the same room and exclusively in the morning and at noon at fixed times. In addition, lifestyle changes and gain or loss of weight can cause volume changes in the facial region. These possibly confounding factors were not controlled for in this study. Because of intervals of many months pre-operatively and of at least six months post-operatively, a slight difference in weight and body condition cannot be completely excluded, although no patient with an extreme weight change was included here. Furthermore, a previous study indicated that individuals who are obese do not experience objective changes in their nasal anatomy [21].

In our rhinoplasty patients, structured taping with steristrips of the nose was performed for two months after removal of the nasal cast. The compliance of the patients can only be assumed and might be another source of error in the evaluation. In general, post-operative taping might contribute to less ecchymosis and reduced periorbital swelling [22].

Our study showed that, between the third and sixth month, some distance decrease still occurs at the defined landmarks. In our daily clinical practice, we inform the patients about a decrease in the swelling of the nose for up to one year. Our findings underline the need for this follow-up. These anatomical landmarks include the points STN, STOR, STML, STMR, NB, and NBP. Septorhinoplasties predominantly affect the NB and NBP points. Therefore, the changes occur most clearly here. The alar region, especially in functional rhinoplasties, is less affected not showing large changes in this region. During the first few months, adjacent regions (STN, STOR, STML, STMR) are also affected. The soft tissue points were specifically selected based on the surgical procedure and the distinct anatomical location. The reproducibility of these soft tissue points is extremely high. The majority of the points can be indicated with an accuracy of up to 1 mm, relatively independent of ethnicity and gender [23]. Low inter- and intra-reader variability was found. Values between 0.5 and 0.75, between 0.75 and 0.9 and above 0.90 indicate moderate, good, and excellent reliability according to the 95% confidence interval of the ICC [24]. The differences in quality are attributable to the variable degrees of certainty of the anatomical landmarks, which are sometimes easier and sometimes more difficult to mark. This leads to variable intraclass coefficients depending on the anatomical landmark. The soft tissue points evaluated in this study have also been tested and confirmed in studies for accuracy and reproducibility [23]. Since any 3D object can be assigned to a coordinate system (x, y, z), the selected points can be reproduced in all planes [23,25]. The raters need a good understanding of the anatomy and to be able to use 3D photography to a sufficient standard to create a reproducible result [23].

The evaluation of the outcome of septorhinoplasties can be improved by 3D imaging in the future, as the surgeon thus has an additional tool to evaluate the result [26]. In addition, the patient must be informed about their surgery and can therefore be made aware of what to expect in terms of swelling and soft tissue changes over a period of time [1]. One previous study involved an evaluation of the differences of the swelling behavior in milliliters, i.e., as a volume [27]. In 2019, another study [5] was published that also considered such volume changes to septorhinoplasties in order to investigate the post-operative resolution of swelling after rhinoplasty. Patients were closely monitored by using 3D images taken at various times from five days to over 250 days after surgery. The nose experienced the greatest volume swelling between the 7th and 14th day after surgery. Unfortunately, these time points cannot be addressed in our patients as they are still wearing the external cast. Over time, an average volume loss of 2.8 mL ± 0.7 was previously observed, ranging from seven days post-surgery to well over 250 days [5]. In terms of swelling distribution, the upper two thirds of the nose consistently showed more swelling than the tip of the nose in that earlier investigation. In our study, points NBP and NB were also exposed to significant alterations, whereas PN showed no significant changes. In areas in which minimal swelling obscured delicate features, swelling was more prominent in the tip of the nose, although its total volume was less affected than the upper two-thirds of the nose [5].

In our study, we limited ourselves to evaluation and comparison by using surface distance maps and individual distance measurements, because an evaluation of the volumes would not have been possible with the software used and without unilateral cropping and filling of the facial scans. Filling, for example, of the nostrils, would have made the evaluation inconsistent and statements of volume changes unrepresentative. The operation was performed in a standardized manner by the same surgeon and according to an identical surgical protocol in each case. Nevertheless, the use of different surgical techniques depending on the pathology might lead to a slight variation during and after surgery and might influence the post-operative swelling behavior differently. We did not use a piezotome in our patients for a better comparability of the results. The comparison of structural versus preservation rhinoplasty by using the piezotome would be an interesting subject for further studies. A similar study performed on patients undergoing bimaxillary orthognathic surgery showed that the greatest volume loss occurs between the first post-operative week and the first post-operative month [28]. However, swelling can still be measurable between the 6th and 12th post-operative months [29]. Reategui et al. reported that, in bimaxillary orthognathic surgery patients after 1 year, an estimated 10% of the initial facial swelling is still present [30]. In orthognathic surgery, 90% of the final volume is regained after approximately 3 months [31]. Surface distance maps have been created to visualize swelling behavior, since such maps are suitable for recording changes over time [32]. With this procedure, all surgical interventions can be compared pre- and post-operatively and related to the desired effect. Surgeons can thus demonstrate to the patient objectively those areas that have changed and the extent of those changes.

The color-coded maps are particularly useful for recording soft tissue changes, which are more difficult to evaluate than bony changes [12]. Changes in distances between the two scans are also color-coded, where, for example, red stands for areas that are further apart [25].

Of course, 3D photographic systems still play a minor role in current clinical practice. The gold standard remains pre-operative 2D photography [1,33]. An optimal 3D photography system has not yet been created. The most important aspect is that a system can be used quickly, is repeatable, and does not impose any health restrictions on the patient [34]. Analysis of not only facial shape, but also color and skin texture, teeth, and muscle shape is advantageous [34]. Following previous studies by our group (data not yet published), we decided to use the Artec Space Spider Camera, since it produced significantly better results in terms of accuracy compared with two other systems (Planmeca ProFace^®^ and Bellus3D Dental Pro application^®^).

The use of 3D photography has the benefit of making surgeons more efficient, operations safer, and anesthesia time shorter [34]. The surgical method can be evaluated depending on the underlying pathology with subsequent improvement in patient care [34]. Nevertheless, stationary and mobile camera systems remain expensive; mobile applications on smartphones might save costs in the future and thus lead to an increased use of 3D photography [35]. The handling and protection of sensitive patient-related data will, however, remain a challenge [35].

The limitations of our study include a smaller number of participants, as the evaluation is really time consuming, a lack of active comparison, and the short duration of follow-up (6 months). Further studies should be made in the future with the experience from now [6].

## 5. Conclusions

The results from this study revealed that 3D scans show significant changes in various anatomical landmarks over time and provide valuable insights into the post-operative recovery process following septorhinoplasties.

## Figures and Tables

**Table 1:** Characteristics of all 29 patients.

Variable	n (%)
Female	20 (69)
Male	9 (31)
Age, years (mean ± SD)	34.2 ± 14.6
**Diagnosis/intention**	**n (%)**
Aesthetically + functional	29 (100)

**Table 2:** Significant differences between the mentioned pairs and landmarks.

Paired Samples Test										
		Paired Differences					t	df	Significance	
		Mean	Std. Deviation	Std. Error Mean	95% Confidence Interval				One-Sided *p*	Two-Sided *p*
					Lower	Upper				
Pair 1	STN—pre/1 month vs. STN—pre/3 months	0.217	0.508	0.141	−0.090	0.523	1.538	12	0.075	0.150
Pair 2	STN—pre/3 months–STN—pre/6 months	0.172	0.384	0.077	0.013	0.330	2.231	24	0.018	0.035
Pair 3	STN—1/3 months–STN—3/6 months	0.026	0.638	0.213	−0.465	0.517	0.122	8	0.453	0.906
**Paired Samples Test**										
		**Paired Differences**					**t**	**df**	**Significance**	
		**Mean**	**Std.** **Deviation**	**Std. Error Mean**	**95% Confidence Interval of the Difference**				**One-Sided *p***	**Two-Sided *p***
					**Lower**	**Upper**				
Pair 1	STOR—pre/1 month vs. STOR—pre/3 months	−0.312	0.384	0.107	−0.544	−0.080	−2.930	12	0.006	0.013
Pair 2	STOR—pre/3 months vs. STOR—pre/6 months	0.030	0.487	0.097	−0.171	0.231	0.311	24	0.379	0.758
Pair 3	STOR—1/3 months vs. STOR—3/6 months	0.277	0.452	0.151	−0.070	0.624	1.840	8	0.052	0.103
Pair 1	STML—pre/1 month vs. STML—pre/3 months	0.541	0.708	0.196	0.113	0.968	2.755	12	0.009	0.017
Pair 2	STML—pre/3 months vs. STML—pre/6 months	0.105	0.404	0.081	−0.061	0.272	1.304	24	0.102	0.205
Pair 3	STML—1/3 months vs. STML—3/6 months	−0.431	0.700	0.233	−0.969	0.107	−1.847	8	0.051	0.102
Pair 1	STMR—pre/1 month vs. STMR—pre/3 months	0.621	0.487	0.135	0.327	0.916	4.595	12	0.000	0.001
Pair 2	STMR—pre/3 months vs. STMR—pre/6 months	0.130	0.380	0.076	−0.027	0.287	1.714	24	0.050	0.099
Pair 3	STMR—1/3 months vs. STMR—3/6 months	−0.809	0.337	0.112	−1.068	−0.550	−7.197	8	0.000	0.000
Pair 1	NB—pre/1 month vs. NB—pre/3 months	0.986	0.547	0.152	0.656	1.317	6.501	12	0.000	0.000
Pair 2	NB—pre/3 months vs. NB—pre/6 months	0.149	0.382	0.076	−0.009	0.306	1.946	24	0.032	0.063
Pair 3	NB—1/3 months vs. NB—3/6 months	−1.075	0.528	0.176	−1.481	−0.669	−6.109	8	0.000	0.000
Pair 1	NBP—pre/1 month vs. NBP—pre/3 months	1.296	0.825	0.229	0.798	1.795	5.667	12	0.000	0.000
Pair 2	NBP—pre/3 months vs. NBP—pre/6 months	0.232	0.507	0.101	0.023	0.441	2.286	24	0.016	0.031
Pair 3	NBP—1/3 months vs. NBP—3/6 months	−1.035	0.575	0.192	−1.477	−0.594	−5.406	8	0.000	0.001

## Data Availability

The data sets obtained and analyzed in the current study are available from the corresponding author upon reasonable request.
